# Bone Cement Implantation Syndrome: A Rare Disaster Following Cemented Hip Arthroplasties—Clinical Considerations Supported by Case Studies

**DOI:** 10.3390/jpm13091381

**Published:** 2023-09-15

**Authors:** Flaviu Moldovan

**Affiliations:** Orthopedics-Traumatology Department, Faculty of Medicine, “George Emil Palade” University of Medicine, Pharmacy, Science, and Technology of Targu Mures, 540142 Targu Mures, Romania; flaviu.moldovan@umfst.ro; Tel.: +40-(75)-4671886

**Keywords:** BCIS, polymethyl methacrylate, bone cement, hip arthroplasties

## Abstract

Severe symptoms such as hypoxemia, hypotension, and unexpected loss of consciousness may develop during surgical interventions that use polymethyl methacrylate (PMMA), or as it is commonly known, bone cement. Physicians recognize this amalgam of clinical manifestations more and more as a distinct entity that bears the name of bone cement implantation syndrome (BCIS). Trauma cases, especially hip fractures, are seen to have a higher incidence of developing this complication compared to orthopedic elective ones. This research aims to present a detailed description of six severe BCIS cases in order to raise awareness and to emphasize its importance. Five of them had fatal outcomes, which demonstrate the necessity of future research on this topic, as little is known about it presently. In the Discussion section, a narrative overview from the scientific literature is performed on potential risk factors, prevention measures, and management strategies. The experience gathered through this case series may aid medical staff in the development of diagnostic and therapeutic protocols, thus improving safety when cemented surgical techniques are used on a high-risk group of patients.

## 1. Introduction

Total hip replacement (THR) is considered one of the most effective procedures performed not only in orthopedics but in all surgical fields. Its main indication is end-stage osteoarthritis, providing outstanding results in the management of patients with disabling symptoms such as loss of function, severe pain, and stiffness. As a matter of fact, it is considered “the operation of the century” [1]. In the European Union, there is a rate of over 150 arthroplasties performed per 100,000 inhabitants in one year [2], and the projected volumes are expected to rise considerably by 2030, as suggested by multiple studies [3,4,5].

Osteoporosis represents a major medical condition in the elderly population as it can contribute very frequently to hip fractures through a low-traumatic mechanism [6]. The residual lifetime risk for these injuries is approximately four times higher for women compared to men at the age of 50 [7]. It is estimated that by 2050, the incidence of hip fractures will rise above 6 million [8]. Moreover, frail patients who need to undertake hip replacements represent a high-risk category of morbidity and mortality due to their associated pathological conditions, which is reflected through high ASA (American Society of Anesthesiologists, Schaumburg, IL, USA) scores [9]. Hemiarthroplasty represents an acceptable option for this low-demand group of patients, and it is frequently performed [10]. Although THR may provide the same or even better functional outcome over 24 months, the additional risk of complications needs to be taken into account [11,12]. The biological state of the patient also plays an important role in terms of deciding the fixation method used for the prothesis components. A retrospective cohort study by Yang et al. [13] confirmed that fixation in low-density bone stock of the femoral component with cement has a better HHS (Harris Hip Score) and revision rate. Fernadez et al. [14] reported an increased quality of life and lower risks of periprosthetic fractures by using cemented hemiarthroplasties in older patients. It is also well known that hybrid or cemented fixation is much more cost-effective in both male and females no matter the age group [15,16]. 

A more scientific term for bone cement is polymethyl methacrylate (PMMA). This acrylic composite is formed from an exothermic reaction (polymerization) by mixing a powder and a liquid component in a 2 to 1 ratio. Surprisingly, this polymer has no adhesive properties as it relies on firm mechanical interlocking between the irregular interstices of the bone surface and the implant, thus acting like space filler [17]. Radiopaque agents are added in the powder composite for easy follow-up of the eventual prosthetic components’ loosening or osteolysis processes [18]. Joint replacement implants are prone to bacterial growth, which may multiply depending on the immune status of the organism and cause unwanted complications. As a consequence, antibiotic-loaded cements were developed, conferring the advantage of local action compared to systemic administration (Table 1) [19]. This type of cement enhanced with antibiotics has been used not only for arthroplasties but also trauma cases. For example, the Masquelet technique involves a staged approach using temporary cement spacers followed by bone grafting in the treatment of posttraumatic defects [20]. Other surgical procedures that rely on this material are vertebroplasties and kyphoplasties for the management of compression fractures of the vertebrae [21]. Different additives have been analyzed in combination with PMMA. Vitamin E in a concentration of 10% was used successfully to boost the cytocompatibility and reduce the peak temperatures. Silver and chitosan nanoparticles showed their antibacterial activity, as well as nano-MgO (12.8 nm) and nano-BaSO4 (100 nm), which promoted osteoblast adhesion and reduced tissue necrosis [22,23].

Bone cement implantation syndrome (BCIS) is a sporadic and potentially lethal complication that may manifest both intra- and perioperatively [24]. It is associated in most cases with hip and knee arthroplasties, although unique occurrences have been presented in the past. Qin et al. [25] provided such an example where they treated a man suffering from chronic osteomyelitis in his tibia with antibiotic-loaded cement. The current scientific literature does not provide a clear definition, but anesthesiologist and orthopedic surgeons recognize it more and more as a distinct entity. This syndrome combines a number of clinical manifestations such as hypoxia, systemic hypotension, arrhythmias, high pulmonary vascular resistance (PVR), unconsciousness, and myocardial infarction [26,27,28,29]. Donaldson et al. [30] were the first to suggest a severity classification that is still of reference presently (Table 2).

The objective of the present research is to raise awareness and provide a comprehensive narrative overview of what is known in the scientific literature about BCIS. It may also set the groundwork for future clinical studies and be of use for educational purposes as it includes a case series that can aid medical staff in the early identification and management of this complication.

## 2. Clinical Cases

### 2.1. Case 1 

A 49-year-old female patient presented to the Emergency Department with pain and loss of function in her left lower limb, caused by a fall from the same level, an event that occurred 12 days ago. During this period, she did not use anticoagulation medication. After clinical and paraclinical investigations, the patient was admitted to the Orthopedics—Traumatology Department with the diagnosis of left femoral neck fracture (FNF) Garden type IV, chronic alcoholism. Other than that, her medical history was unexceptional.

Five days later, after a proper preoperative and anesthesiologic preparation, a cemented total hip arthroplasty of the left hip was performed under intraspinal regional anesthesia with 10 mg of 0.5% heavy (H) marcaine and 5 µg of sufentanyl. During the implantation of the femoral stem, the patient became disoriented, tachypneic, and showed pulseless electrical activity (PEA). Resuscitation maneuvers were initiated according to the protocol (CPR, airway protection through intubation; ventilation with self-inflating balloon). After 29 min, her sinus rhythm was reverted.

Postoperatively, the patient was transferred from the orthopedic operating room to the Intensive Care Unit (ICU) in an altered general condition, presenting: symmetrical reactive pupils, a Glasgow Coma Score (GCS) of 7 points, endotracheal intubated and mechanically ventilated, fraction of inspired oxygen (FiO_2_) 100%, oxygen saturation (SpO_2_) 66%, hemodynamically unstable with a blood pressure (BP) of 61/33 mmHg, and a heart rate (HR) of 133 beats per minute without inotropic and vasoactive support. Shortly after, she presented an episode of ventricular tachycardia without a pulse, the reason for which external electric shock (200 joule) was administered successfully.

A cardiological examination was requested, raising the suspicion of pulmonary embolism (PE). As a matter of urgency, the patient was shifted to the Interventional Cardiology Department for a catheter-directed thrombolysis (CDT) of the left pulmonary artery. Post-procedure, the patient was still hemodynamically unstable on support with norepinephrine and dobutamine, blood pressure (BP) of 79/40 mmHg, and a heart rate (HR) of 118 beats per minute. Forty-four minutes later, on the ICU, an episode of extreme bradycardia up to asystole occurred. Cardiopulmonary resuscitation (CPR) was started immediately and continued according to the Advanced Cardiovascular Life Support (ACLS) protocol for approximately 30 min, when time of death was declared.

### 2.2. Case 2 

An 89-year-old female patient with right femoral neck fracture (FNF) garden type IV was admitted to the Orthopedics—Traumatology Department after refusing surgical treatment and hospitalization 7 days ago. The medical history and cardiological preoperative exam revealed that she also had chronic heart failure (CHF) with mildly reduced ejection fraction (45%), mild mitral insufficiency, hypertensive cardiomyopathy, high blood pressure (HBP) stage 2, persistent atrial fibrillation (AFib), right bundle branch block (RBBB), chronic venous insufficiency (CVI), and prerenal acute kidney injury (AKI). Her most notable chronic medication included angiotensin converting enzyme (ACE) inhibitors (enalapril, 5 mg, Enap^®^, Hyderabad, India) and vasodilators (pentoxifylline, 400 mg).

Two days later, after a proper preoperative and anesthesiologic preparation, a cemented bipolar hemiarthroplasty of the right hip was performed under intraspinal regional anesthesia with 15 mg of 0.5% heavy (H) marcaine and 5 µg of sufentanyl.

Immediately after surgery, the patient became desaturated, conscious but difficult to cooperate with, and disorientated temporally and spatially (CGS 13 points), the reason for which she was transferred to the ICU. There, her general condition worsened, the patient became unresponsive and tachypneic, needing intubation with a size 7 endotracheal cannula.

After 19 min, an episode of extreme bradycardia occurred. CPR was started and Epinephrine 2 mg i.v. together with Atropine 1 mg i.v. were administered, her sinus rhythm being successfully reverted. A 7F_2_ triple-lumen central venous catheter (CVC) on the right internal jugular vein was mounted. Shortly after, the patient presented cardiac arrest, which was fatal despite the attempted resuscitation maneuvers and administration of Epinephrine 7 mg i.v.

### 2.3. Case 3

**Figure 1:**
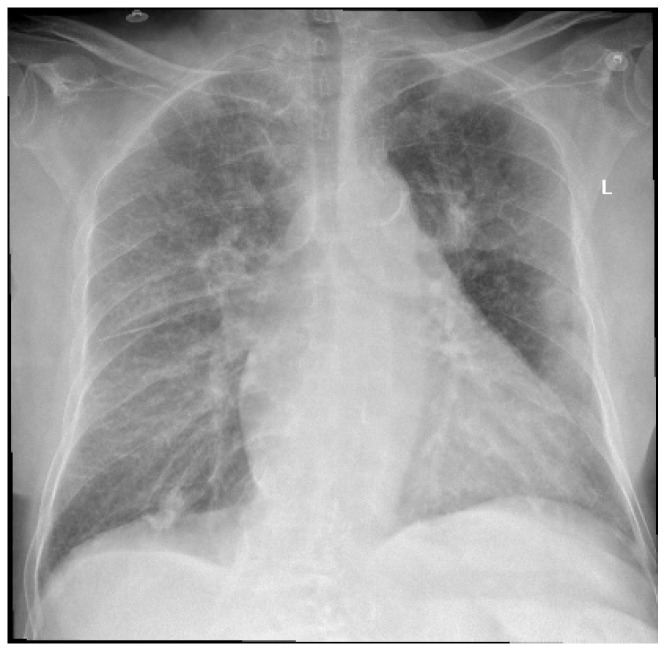
Chest X-ray at admission—describing small left pleural effusion; bilateral diffuse reticule-micronodular interstitial lung pattern; questionable opacities at the level of the posterior left VI costal arch and right intercostal XI space; pulmonary vascular congestion; global enlarged heart; atherosclerotic aorta. L—left side.

An 83-year-old female patient was admitted to the Orthopedics—Traumatology Department with a left femoral neck fracture (FNF) garden type IV due to a fall from the same height. Upon preoperative examination, the following associated comorbidities were diagnosed: bronchopneumonia (Figure 1), chronic ischemic heart disease (IHD), high blood pressure (HBP) stage 2, left ventricular failure NYHA (New York Heart Association) type 2 with a left ventricle ejection fraction (LVEF) of 50%, moderate aortic valve stenosis (mean gradient 34 mmHg, aortic valve area 10 mm), tricuspid regurgitation grade 2, and permanent atrial fibrillation (AFib), for which she was taking oral anticoagulants (rivaroxaban, 20 mg, Xarelto^®^, Leverkusen, Germany), diuretics (spironolactone/furosemide 50 mg/20 mg, Diurex^®^, Niles, IL, USA), and beta-blockers (nebivolol, 5 mg, Nebilet^®^, New York, NY, USA).

Due to the pulmonary symptoms, antibiotic therapy and oxygen therapy were administered. The surgical intervention was postponed for 14 days when the patient underwent cemented bipolar hemiarthroplasty of the left hip. Intraspinal regional anesthesia with 10 mg of 0.5% heavy (H) marcaine and 5 µg of sufentanyl was administered. After inserting the cement into the femoral canal, the patient developed an episode of extreme bradycardia up to pulseless electrical activity (PEA). The surgeons stopped the procedure and resuscitation maneuvers started according to the protocol (CPR, endotracheal intubation with mechanical ventilation and administration of Epinephrine 2 mg i.v.) for 5 min, when her sinus rhythm was reverted with blood pressure (BP) of 190/134 mmHg and a heart rate (HR) of 180 beats per minute.

Postoperatively, the patient was shifted to the ICU, without reversion of anesthesia, in critical general condition (GCS 5 points), hemodynamically unstable on double inotropic and vasoactive support, with blood pressure (BP) of 119/69 mmHg and a heart rate (HR) of 194 beats per minute. The next day, safe extubating was decided, and spontaneous breaths were present with the supplementation of oxygen (11 L/min) through a beathing mask. After 6 days in the ICU, she was transferred back to the Orthopedics—Traumatology Department and discharged in a good general condition a couple of days later.

### 2.4. Case 4

An 86-year-old male patient with a left femoral neck fracture (FNF) garden type III was admitted under an emergency regime to the Orthopedics—Traumatology Department. His medical history included: chronic heart failure (CHF), chronic kidney disease (CKD) stage IV, bronchial asthma, and iron deficiency anemia, all of which were under treatment. Ecocardiography revealed mild aortic valve stenosis and a severely reduced ejection fraction (25–30%), and the electrocardiogram showed left anterior fascicular block (LAFB). 

A cemented total hip arthroplasty was performed three days later under intraspinal regional anesthesia with 10 mg of 0.5% heavy (H) marcaine. After a reduction in the prothesis, the patient developed an episode of extreme bradycardia (28 beats per minute). He was administered 1 mg of Atropine, 240 mg of Aminophylline, and 3 × 20 mg Ephedrine, after which intubation with a size 8 endotracheal cannula was decided. A total of 10 mg of Epinephrine was administered, but despite resuscitation maneuvers, the patient could not be retrieved.

### 2.5. Case 5

**Figure 2:**
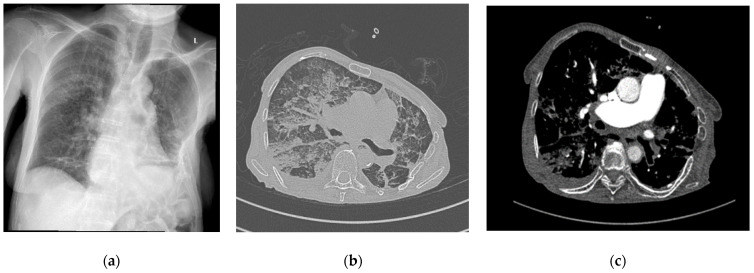
(**a**) Chest X-ray at admission; (**b**) contrast-enhanced chest CT scan lung window after surgery; (**c**) contrast-enhanced chest CT scan soft tissue window after surgery. L—left side.

A 70-year-old female patient with right femoral neck fracture (FNF) garden type III returned to hospital after she refused surgical treatment 6 days ago. She was also suffering from pulmonary fibrosis (Figure 2a), rheumatoid arthritis (RA), chronic venous insufficiency (CVI), and high blood pressure (HBP) stage 1.

The same day, a cemented total hip arthroplasty was performed under intraspinal regional anesthesia with 10 mg of 0.5% heavy (H) marcaine and 5 µg of sufentanyl. While the cement was digitally applied into the femoral canal, the patient presented an emetic episode and tachycardia arrhythmia. After the surgery, she was agitated and desaturated. CT angiography (CTA) was requested that excluded pulmonary embolism (PE), describing bilateral bronchopneumonia outbreaks, pulmonary fibrosis associated with bronchiectasis, and pulmonary hypertension, with minimal pleurisy (Figure 2b,c).

Approximately 3 h later, the patient developed cardiac arrest. BLS resuscitation maneuvers were started by the physicians on the ward and continued as ALS according to the protocol by the emergency team. A total of 11 mg of Epinephrine (1 mg every 3–5 min) was administered and 500 mL sodium chloride 0.9% i.v. infusion. The patient remained unresponsive, and 43 min later she was declared dead.

### 2.6. Case 6

An 86-year-old female patient with right femoral neck fracture (FNF) garden type III was admitted for surgical treatment following a fall from the same height. She was not taking any chronic medication despite having high blood pressure (HBP) stage 2, chronic heart failure (CHF), and hypercholesterolemia. The laboratory data showed hypopotassemia (3.17 mmol/L), which was corrected at the Emergency Department. 

A cemented bipolar hemiarthroplasty was performed under orotracheal intubation and general anesthesia with 150 mg of ketamine and 40 mg of esmeron. Postoperatively, the patient was respiratory and hemodynamically stable but difficult to cooperate with and unresponsive to verbal stimuli. The suspicion of a stroke was raised, and a neurological evaluation was requested. The consultant confirmed that she had mixed aphasia and right hemiplegia, requiring an emergency brain CT. 

The vital signs were degrading (BP 78/43 mmHg, HR 128 beats per minute, SpO_2_ 84%), so a cardiological evaluation was requested due to the low cardiac output. Following the consultation, Norepinephrine support was started, and acute pulmonary embolism (PE) suspicion was raised, requiring a CT angiography (CTA). Shortly after the consults, the patient developed a cardiac arrest and could not be saved despite all efforts made by the physicians.

All cases presented in this study were obtained from the Orthopedics—Traumatology Department of Mures County Emergency Hospital in Targu Mures, Romania. Table 3 presents an overview of the six cases described previously.

## 3. Discussion

BCIS may be one of the most unrecognized complications due to its wide variety of clinical manifestations that can present in different grades of severity. The clinical cases described in the current scientific literature have a tendency to present just casualties as physicians easily overlook milder forms. Olsen et al. [31], for example, identified an incidence of around 30% in their retrospective study that included over 1000 patients that underwent cemented partial arthroplasties for hip fractures. They have also pointed out that only 5–7% presented a severe grade (II or III). A similar overall percent of incidence was reported by Rassir et al. [32], but they included different types of joint replacement surgeries: 31% (282/915) in partial hip arthroplasties, 28% (210/765) in total knee replacements, 24% (165/677) in total hip replacements, 23% (47/206) in revision arthroplasties, 20% (113/558) in partial knee replacements, and 16% (28/173) in shoulder arthroplasties. 

In our department, no scheduled orthopedic intervention presented this accident. A recent study [33] suggested that this syndrome is six times more frequent in trauma cases than in elective surgeries (0.71% compared to 0.12%), and, more important, mortality was 10 times higher (0.17% compared to 0.017%, *p* < 0.001). It is clear that the biologic state of the individuals together with the associated comorbidities play an important role in triggering the pathophysiological mechanisms. Bony metastases generate a highly vascular and permeable bone surface, thus creating a hypercoagulable state, and procoagulants like fibrinopeptide-A are released by malignant cells, both of which considerably raise the risk of thromboembolism [34,35,36]. Porotic bone and extracapsular hip fractures can produce a similar predisposition [30]. Five of our six cases were females, and it is well known that osteoporosis has a higher incidence in this group, though no gender correlation with the syndrome was demonstrated. As for intertrochanteric fractures, they produce a higher blood loss compared to intracapsular hip fractures, producing a higher chance of an embolic event occurring [37,38]. Schwarzkopf et al. [39] implied that elderly patients and those with lung metastases or lung cancer due to decreased lung function are prone to developing BCIS during hip surgery. Cardiopulmonary pathologies like atrial fibrillation (AFib), chronic heart failure (CHF), chronic obstructive pulmonary disease (COPD) with high PVR, and pulmonary hypertension play a role in hypoxia, blood acidosis, and the systemic inflammatory response [31]. A future research direction deducted from our cases that would be of interest to explore is the relation with the grade of aortic stenosis and other pathologies, like pulmonary fibrosis and bronchopneumonia. Chronic medications, such as beta-adrenergic blockers, diuretics, oral anticoagulants, and angiotensin-converting enzyme (ACE) inhibitors, were linked with the syndrome [31]. It is clear from this case series that patients who initially refused and postponed their hospitalization several days were predisposed for such an event despite proper preoperative and anesthesiologic preparation prior to surgery, and this aspect should also be taken into consideration.

The pathophysiological mechanism behind BCIS is not yet fully established. At first, the hemodynamic instability was attributed to methyl-methacrylate monomer toxicity that can cause the direct relaxation of arterial and venous smooth muscles. This theory was abandoned due to extremely high MMA concentrations needed in humans in order to produce any hemodynamic alterations [40,41]. Recently, the embolus-mediated model was advocated. The mechanical consequences which appear during femoral stem insertion combined with the exothermic expansion of cement as marrow, platelets, fibrin, air, bone, and cement debris can enter circulation due to high intramedullary pressure (more than 300 mmHg). The emboli thereby formed migrates to the right side of the heart, pulmonary circulation, or cerebral circulation, thus producing obstruction. Vasoconstriction may be produced also by the mediators released by the emboli itself, such as Thrombin and Thromboplastin, or by the endothelial damage it produces through Endothelin-1 and 6-keto prostaglandin-F1 alpha. The two etiologies in this model are suggested to be responsible for the hypoxemia as there is a ventilation perfusion (V/Q) imbalance and also hypotension due to increased PVR that reduced in left ventricular compliance, filling, and cardiac output (CO) [30,42]. Other proposed factors include the complement-peptides C3a and C5a that are known to have vasoconstrictor and bronchoconstrictor properties [43], and the plasma histamine release that can produce hypotension, as in anaphylaxis. As a matter of fact, prophylaxis with 4 mg Cleastine and 400 mg Cimetidine (H1, H2–antagonists) i.v. 15 min prior to cementation is beneficial [44]. What is certain is that none of the above mechanisms explain all the questions; as a matter of fact, a multi-modal theory was suggested that takes into account all the factors associated with the patient [45].

Both the anesthesiologist and orthopedic surgeon play a major role in preventing this catastrophic complication by identifying high-risk patients and tacking all the necessary measures. One strategy is to use, from the beginning, an uncemented implant as some modern designs present a lower incidence of periprosthetic fracture and are more suitable for poor bone stock [46]. It was demonstrated that long and shape-closed design femoral components along with primary arthroplasties where the intramedullary canal was not yet reamed are additional contributing factors [47,48,49]. Other preventive surgical techniques include proper lavage of the intramedullary canal in order to remove debris, achieving hemostasis, and drilling a venting hole in the distal femoral shaft in order to minimize pressure and air entrapping when the stem is inserted [50,51]. The type of cement and the way it is placed are also important. Rothberg et al. [35] have concluded in their study that low-viscosity cement does not interfere with the pullout strength of the bone–cement–stem interface and reduces intramedullary pressure compared to high-viscosity cement. The bone-vacuum cementing technique lowers the cement porosity, thus reducing the embolic load of both volatile vasoactive and mechanical compounds [52]. Ultimately, the retrograde insertion of cement with the use of a specific gun produces a uniform increase in pressure [53].

In lower limb surgery, both general and intraspinal regional anesthesia are viable options, both having their own advantages and disadvantages. Currently, there are no studies that favor one technique to the detriment of the other in cemented hip replacement. Instead, high anesthetic vapor concentrations have a negative hemodynamic effect, and nitrous oxide can produce air embolism exacerbation [42]. The early identification of BCIS can be suspected after an instant drop in end-tidal carbon dioxide (ETCO2) values. Thus, intraoperative CO tracking in the form of non-invasive trans-esophageal Doppler monitoring and PiCCO (arterial pulse contour analysis) or a more invasive pulmonary artery flotation catheter are recommended in high-risk patients [45]. Appropriate circulating volumes and arterial pressures before cementation are mandatory. The medical staff should be very vigilant during cementation, femoral stem insertion, joint reduction, tourniquet deflation, if used, and also immediately after the surgery in order to prevent the onset of symptoms [30]. Basic principles of resuscitation are recommended in case of any suspicion. Inspired oxygen concentration must be at 100% and the airway secured. For cardiovascular collapse, the use of aggressive i.v. fluids, pulmonary vasodilators, inotropes, and vasopressors (through a central venous catheter) to support right cardiac contractility are recommended.

There are some situations that may resemble BCIS; therefore, it is important to make a differential diagnosis. BCIS implies pulmonary embolization from the intramedullary contents during arthroplasties as all cases are reported in the perioperative and intraoperative period. PE due to DVT (deep vein thrombosis) is known to occur much later in the postoperative period. For example, Bjørnarå et al. [54] reported a mean time for symptomatic DVT and PE of 21 and 34 days, respectively, after hip arthroplasties. The same differential diagnosis could be made with an ischemic stroke. Emboli formed in BCIS can migrate also cerebral circulation, presenting neurological symptoms at the time of surgery. As noted by Haynes et al. [55], there is a 0.09% risk of developing a postoperative stroke 30 days after a THA or TKA. Anaphylactic shock should also be excluded as patients can develop a disproportionate response to PMMA, especially if they are diagnosed with mastocytosis [56].

The first limitation of this study is regarding its design as this case series offers little basis for generalization of the results to a wider population. Secondly, the incidence of this complication may be much higher than expected because milder forms could have been easily overlooked by physicians. The cases presented were all BCIS grade 3 with severe symptoms, the majority resulting in a fatal outcome. Finally, there is an observer bias as the future research directions and aspects related to the cases could be interpreted in a different manner by the readers.

## 4. Conclusions

BCIS can lead to a cascade of severe manifestations with potential lethal consequences and poor treatment response. Clinicians should be aware at all times of its existence during cemented procedures as this may make the difference between life and death. Further studies that can explain the mechanisms behind this complication and the factors that may trigger it are mandatory for prevention or early diagnosis. This may also contribute to the development of effective management protocols and strategies that can assure a safe outcome for a high-risk group of patients.

## Figures and Tables

**Table 1:** Detailed components of a basic bone cement.

Powder Components	Liquid Components
Co-polymer: polymethyl methacrylate	Monomer: methyl-methacrylate (MMA)
Initiator: benzoyl peroxide (BPO)	Accelerators: N, N dimethyl-p-toluidine (DMPT)
Radiopaque agents: barium sulphate (BaSO_4_) or zirconium dioxide (ZrO_2_)	Inhibitors/Stabilizers: hidroquinone
+/− Antibiotics: gentamicin sulphate, vancomycin, tobramycin, etc.	

**Table 2:** Severity classification of BCIS.

Grade 1	Grade 2	Grade 3
Mild hypoxia (SpO_2_ < 94%) or systemic hypotension with decrease in SBP > 20%	Severe hypoxia (SpO_2_ < 88%) or systemic hypotension with a decrease in SBP > 40% or unexpected loss of consciousness	Cardiovascular collapse necessitating CPR

Abbreviations: SpO_2_—oxygen saturation; SBP—systolic blood pressure; CPR—cardiopulmonary resuscitation.

**Table 3:** Overview of the cases.

	Patient 1	Patient 2	Patient 3	Patient 4	Patient 5	Patient 6
Baseline characteristics						
Age	49	89	83	86	70	86
Sex	Female	Female	Female	Male	Female	Female
BMI	19.2	28.9	21.6	23.9	23.4	25.0
Surgery related data						
Type of anesthesia	Intraspinal	Intraspinal	Intraspinal	Intraspinal	Intraspinal	General
ASA score	III	III	IV	IV	II	IV
Preoperative stay (days)	17	9	14	3	6	1
Duration of surgery (minutes)	80	55	43	85	67	45
Implant characteristics						
Type of prothesis	cTHA	cBHA	cBHA	cTHA	cTHA	cBHA
Type of stem	Taperloc Biomet, 7.5 × 135 mm	Exacta Plus Permedica, Size 6 (145 mm)	PAVI Goupe Lepine, Size 14	Logica Standard Limacorporate Size 4 (156 mm)	Taperloc Biomet, 12.5 × 145 mm	PAVI Goupe Lepine, Size 13
Type of cement	Simplex P Stryker	Surgival with Gentamicin	Surgival with Gentamicin	Simplex P Stryker	Palamed with Gentamicin	Surgival with Gentamicin

## Data Availability

The data used in this study can be requested from the corresponding author.
